# An Advanced TiAl Alloy for High-Performance Racing Applications

**DOI:** 10.3390/ma13214720

**Published:** 2020-10-22

**Authors:** Michael Burtscher, Thomas Klein, Janny Lindemann, Oliver Lehmann, Holger Fellmann, Volker Güther, Helmut Clemens, Svea Mayer

**Affiliations:** 1 Department of Materials Science, Montanuniversität Leoben, Roseggerstr. 12, 8700 Leoben, Austria; thomas.klein@ait.ac.at (T.K.); helmut.clemens@unileoben.ac.at (H.C.); svea.mayer@unileoben.ac.at (S.M.); 2 GfE Fremat GmbH, Gewerbegebiet Süd 20, 09618 Brand-Erbisdorf, Germany; janny.lindemann@gfe.com; 3 Märkisches Werk GmbH, Haus Heide 21, D-58553 Halver, Germany; o.lehmann@mwh.de (O.L.); dr.h.fellmann@mwh.de (H.F.); 4 GfE Metalle und Materialien GmbH, Höfener Str. 45, 90431 Nuremberg, Germany; volker.guether@gfe.com

**Keywords:** intermetallics, titanium aluminides, grains and interfaces, casting methods, microstructural characterization, electron microscopy, fracture behavior, fatigue

## Abstract

Requirements and strict regulations for high-performance racing applications involve the use of new and innovative lightweight structural materials. Therefore, intermetallic γ-TiAl-based alloys enable new opportunities in the field due to their lower density compared to commonly used Ni-base superalloys. In this study, a β-solidifying TiAl alloy was examined toward its use as structural material for inlet and outlet valves. The nominal composition of the investigated TNM alloy is Ti–43.5Al–4Nb–1Mo–0.1B (in at%), which enables an excellent formability at elevated temperatures due to the presence of bcc β-phase. Different hot-extrusion tests on an industrial scale were conducted on the cast and hot isostatic pressed material to determine the ideal microstructure for the respective racing application. To simulate these operation conditions, hot tensile tests, as well as rotational bending tests, at room temperature were conducted. With a higher degree of deformation, an increasing strength and fatigue limit was obtained, as well as a significant increment of ductility. The fracture surfaces of the rotational bending test specimens were analyzed using scanning electron microscopy, revealing the relationship between crack initiation and microstructural constituents. The results of this study show that the mechanical performance of extruded TiAl material can be tailored via optimizing the degree of hot-extrusion.

## 1. Introduction

Engineering intermetallic γ-TiAl-based alloys are commonly used for high-performance applications, such as racing and aviation, due to their excellent specific mechanical and thermal properties [1,2,3,4]. In general, γ-TiAl alloys exhibit good oxidation resistance and microstructural stability during long-term thermal exposure up to service temperatures of 750 °C and, depending on their microstructure, a good creep behavior [5,6,7]. A major benefit is the low density of about 3900 kg/m^3^ to 4200 kg/m^3^, being approximately half of the density of Ni-base superalloys [8]. Thus, γ-TiAl alloys are able to meet the aim of a higher efficiency of propulsion systems, which results in a reduction of fuel consumption and further, a decrease of CO_2_ emissions [9]. Due to the reduced moving mass, the acceleration behavior of TiAl components is improved, yielding a significant advantage against competing materials [10,11,12]. Thus, beneficial response behavior of engines equipped with high-temperature lightweight TiAl components can be achieved [11]. In the case of racing applications, second generation TiAl alloys were the first to achieve commercial success. In the late 1990s, in Formula 1 racing engines, the TiAl valve material was revolutionary and provided a significant performance advantage that has fundamentally changed the engine designer’s concept for light and heat-resistant valves [9,10,11]. Although the number of racing series with approved intermetallic alloys is limited, the number of users operating with intermetallic γ-TiAl valves has increased significantly over the last 25 years. At the end of the 20th century, third generation TiAl alloys with high amounts of Nb and micro-alloying elements gained importance due to their outstanding mechanical properties and oxidation resistance [13,14,15,16,17]. The process-adapted fourth generation TNM alloy with a nominal composition of Ti-43.5Al-4Nb-1Mo-0.1B (at%) enables a solidification pathway via the disordered β-phase. Thus, a homogeneous and fine-grained microstructure as well as a good deformability at elevated temperature, where, e.g., processes such as hot extrusion, forging, and rolling are carried out, can be achieved as reported in [14,18,19]. This alloy system fundamentally extends the opportunity to reach a broader customer base, by providing a cost-effective product while ensuring long-term sustainability in the evermore cost-sensitive high-performance racing market [20]. In order to meet the already mentioned demands and to withstand the different mechanical load conditions, a homogeneous, fine-grained, and defect-free microstructure is required. Therefore, a new ingot production route was used that provides aviation-grade TNM material for the valve production [20,21]. Hence, the raw material was produced by a combination of vacuum arc remelting (VAR) and induction skull melting (ISM) as comprehensively described in [20].

Within this study, the influence of different hot-deformation ratios on the microstructure and mechanical properties of the TNM alloy is examined. Tensile tests and rotating bending tests were performed to evaluate application-relevant properties of the differently deformed specimens. The major objective of this work is to obtain hot-extrusion parameters to adjust the most promising microstructure and thus the best mechanical properties for high-performance racing applications. This may lead to an enhanced lifetime due to optimized processes, as well as the possibility of a lightweight design of these highly loaded parts.

## 2. Materials and Methods

The TNM material was processed by GfE Metalle und Materialien GmbH, Nuremberg, Germany, using double VAR followed by ISM [20]. For this purpose, the feedstock was melted in a water-cooled copper crucible where the melt is steered in a native skull of solidified TiAl until the whole ingot has liquefied. This allowed an excellent homogenization of the heavy alloying elements such as Nb and Mo [20,21]. Afterwards, the melt was centrifugal cast under vacuum in a rotating mold wheel. Subsequently, hot isostatic pressing (HIP) at 1200 °C and 200 MPa for 3 h allowed the closing of the remaining casting porosity, which also improved the chemical homogeneity. Further processing was carried out by MW Racing Märkisches Werk GmbH, Großbodungen, Germany, employing a single and multi-stage hot-extrusion process to obtain four different degrees of deformation φ. These ratios, including a φ of 0, 0.6, 1.4, and 1.9, are calculated according to Equation (1):

(1) $$\varphi \;=\;\int_{r_{0}}^{r_{1}}\frac{\mathrm{dr}}{r}=\;\ln\frac{r_{1}}{r_{0}}$$

where r_0_ is the starting radius and r_1_ represents the radius after deformation. This relation is displayed within the graphical abstract. In the following, the deformation ratios are given as positive numbers, although extrusion results in a reduction of the radius. The specific processing parameters cannot be disclosed, but [8,15,21] can be used as a guideline. To prevent oxidation, evaporation of Al, and heat loss of the rods during transfer and processing, a steel canning was used. A Mo foil acted as a diffusion barrier between the canning and the TNM ingot. After hot extrusion, a one-step heat treatment (HT) was conducted to achieve a stable microstructure close to thermodynamic equilibrium. Due to legal reasons, the HT parameters cannot be disclosed, however, the used temperature was above the service temperature. The specimens used in this study were electric discharge machined from the cast/HIPed, deformed, and HT material.

The metallographic preparation, including grinding and electrolytic polishing, was performed as described in [22]. The microstructural characterization, as well as fracture surface analysis, was conducted by scanning electron microscopy (SEM) using an Evo50 from Zeiss, Oberkochen, Germany, at an acceleration voltage of 15 kV in secondary electron (SE) or backscattered electron (BSE) mode.

Rotating bending tests were conducted at room temperature (RT) with a Pun Z testing machine of the company Schenck, Darmstadt, Germany. To avoid uncontrolled heating of the sample during the experiment, the test frequency was limited to 100 Hz. The samples were polished to minimize residual stresses in the surface region and, therefore, avoid influences caused by the strong surface sensitivity of this testing method. The surface roughness of the tested specimens exhibited an R_a_ value of 0.20 µm and a R_z_ value of 1.62 µm.

Hardness tests according to Vickers HV10 were carried out using an M4C O25 G3M universal testing machine from Emco-Test, Kuchl, Austria. The values in this work correspond to the mean of at least 5 different measurements and the associated errors are given by the standard deviation. Hot and RT tensile tests were conducted at a starting strain rate of 2.4·10^−5^ s^−1^ at GfE Fremat GmbH, Freiberg, Germany, at RT, 600 °C, 700 °C and 800 °C.

## 3. Results and Discussion

### 3.1. Microstructure

The influence of the microstructure of TiAl alloys on the mechanical properties has been extensively reviewed in the literature, e.g., see [8,18,23,24,25]. It is evident that the grain size is one of the critical parameters for fatigue and tensile strength [26]. Using increasing deformation ratios, the grain size can be decreased significantly, which is mainly attributed to recrystallization leading to a refined and early texture-free microstructure [8,27,28].

**Figure 1:**
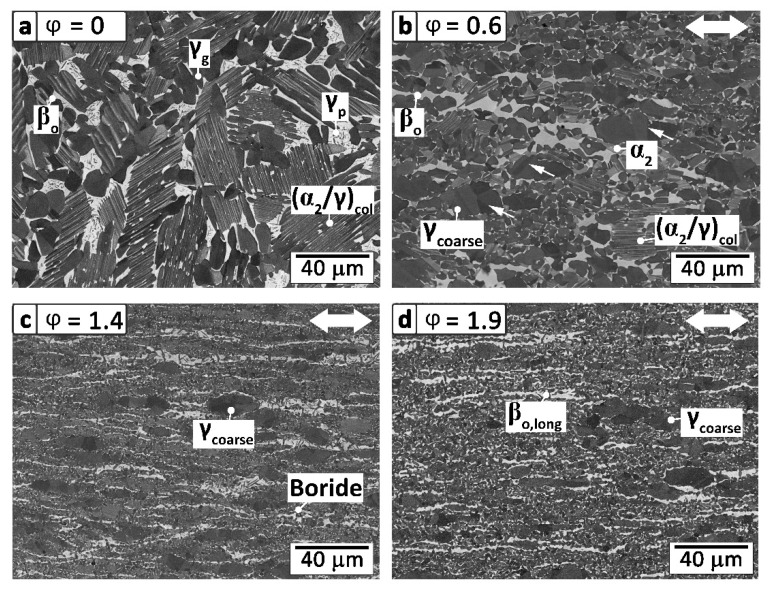
SEM micrographs recorded in backscattered electron (BSE) mode of the microstructures of the different specimens investigated within this work. All specimens were heat treated to enable static recrystallization as well as to provide a thermally stable microstructure. (**a**) Cast/hot isostatic pressed (HIPed) and heat treated (HT) microstructure containing (α_2_/γ)_col_, γ_g_, and surrounding β_o_-phase; (**b**) the φ = 0.6 deformed specimen with partly recrystallized (α_2_/γ)_col_, as well as fine γ and α_2_ grains. The arrows indicate twins within the γ_g_ grains; (**c**) illustrates the microstructure of the φ = 1.4; and (**d**) the φ = 1.9 deformed samples. The latter two exhibit a similar appearance and an almost completely recrystallized microstructure. Solely isolated γ_coarse_-grains and the elongated β_o_-phase constitute deviations from the homogenous microstructure. The extrusion direction is indicated by white arrows in the right upper corner of the SEM images.

In Figure 1a, the initial microstructure of the cast/HIPed and subsequently heat treated material is depicted. The TNM alloy shows coarse α_2_-Ti_3_Al/γ-TiAl colonies ((α_2_/γ)_col_) surrounded by a seam of β_o_-TiAl phase and a high amount of globular γ, γ_g_, grains. These γ_g_ grains exhibit a mean diameter ($\bar{d}$) of 10.7 µm. Within the β_o_ phase, small γ platelets, γ_p_, are visible, which form during the cooling sequence and the subsequent heat treatment [29]. Within the (α_2_/γ)_col_, secondary β_o_-phase precipitates during HIPing or subsequent HT to accomplish thermodynamic equilibrium [30,31]. The material condition shown in Figure 1a exhibits a mean hardness of 342 ± 3 HV10, representing a reasonable value for a cast/HIPed and HT microstructure [8].

In Figure 1b, the microstructure of the hot-extruded (φ = 0.6) material is shown, exhibiting a radial deformation degree of 0.6. The β_o_-phase appears elongated in loading direction, which indicates a good deformability of the disordered body-centered cubic (bcc) β-phase at deformation temperatures [15,21]. A major part of the microstructure consists of fine equiaxed globular α_2_- and γ-grains. However, a small number of (α_2_/γ)_col_ remains and their lamellae are orientated in the extrusion direction [32]. Due to the applied deformation energy during the hot-extrusion, the disordered hexagonal α-phase and γ-laths of the colonies are subjected to a strong driving force for recrystallization [32]. Colonies arranged lengthwise or nearly lengthwise to the axis of the ingot are not deformed as much as their twisted counterparts [33,34]. Hence, the driving force for recrystallization is lower, reasoning the occurrence of single (α_2_/γ)_col_, which do not undergo recrystallization [33]. Similarly to this phenomenon, coarse γ-grains elongated in the deformation direction can be noticed. Within these grains, deformation twins are visible, which are marked by white arrows in Figure 1b. The mean size of these γ_g_ grains is 6.9 µm and the hardness of this type of microstructure is 356 ± 3 HV10. Compared to the cast/HIPed and HT state, a higher hardness due to the smaller grain size is achieved [35,36].

In Figure 1c, the microstructure of the double-extruded sample with φ = 1.4 is shown. The increased degree of radial deformation leads to a finer and even more homogenous microstructure. Except for a few coarse γ (γ_coarse_) grains, the whole material is recrystallized [32]. Furthermore, elongated β_o_-grains are labeled as β_o, long_, which indicate the deformation direction. The average grain size of the globular γ-grains was reduced to 1.8 μm. In summary, persistent γ_coarse_-grains surrounded by a fine matrix of α_2_- and γ-grains, as well as elongated β_o_-grains, can be found. Their occurrence is explained by the growth and agglomeration of globular γ-grains during the HIP treatment [8,21,37]. During hot-deformation, these γ-grains are elongated and partly recrystallized [32,38]. Boron additions to the alloying system result in the formation of Ti-borides during solidification [39,40]. In the case of the TNM alloy, only monoborides have been detected. These borides act as heterogeneous nucleation sites during solidification and the solid-state transformation from β- to α-phase [41]. Hence, boron as a minor alloying element effectively reduces the grain size of the cast microstructure [39,40]. During hot deformation, these borides are fractured, as shown in Figure 1c, and act as nucleation sites for recrystallization and, thereby, facilitate the microstructural refinement [42,43].

In Figure 1d, the microstructure of the strongest deformed specimen with φ = 1.9 is depicted, which is very similar to the material with φ = 1.4, as shown in Figure 1c. These two conditions furthermore exhibit a comparable hardness, which amounts to 393 ± 2 and 386 ± 2 HV10 in case of φ = 1.4 and φ = 1.9, respectively. A mean grain size of the globular γ-grains of approximately 1.8 µm is observed at φ = 1.9, indicating that using double extrusion no further refinement occurred by increasing φ from 1.4 to 1.9.

### 3.2. Room Temperature and Hot Tensile Tests

**Figure 2:**
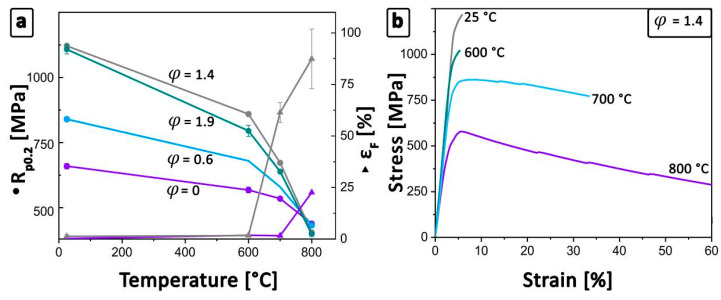
Results of the tensile tests conducted on different material conditions. (**a**) R_p0.2_ as a function of temperature of the as-HIPed, hot-extruded, and HT material from RT to 800 °C. Test values at 600 °C and 700 °C of the φ = 0.6 series are missing due to a lack of sample material. (**b**) Representative σ–ε curves of the material with φ = 1.4 at temperatures from RT to 800 °C. The results of the tensile and tests for all temperatures and material conditions are summarized in Table 1. The error bars indicate the standard deviation of the respective data point, as shown in Table 1.

Tensile testing was conducted at RT, 600 °C, 700 °C, and 800 °C. The obtained results are summarized in Table 1. Figure 2a shows the yield strength (R_p0.2_) in dependence of temperature and deformation ratio of the differently hot-extruded conditions. From RT to 800 °C, the deformed specimens exhibit a clear reduction of the R_p0.2_ and ultimate tensile strength (UTS). The UTS of the undeformed condition behaves contrary to the measured R_p0.2_, as shown in Table 1, and exhibits a moderate increase followed by a decrease at 700 °C and 800 °C. This effect is attributed to the higher elongation and, therefore, a higher UTS is obtained. The following decrease with increasing temperature of the UTS as well as R_p0.2_ in all conditions is ascribed to the higher mobility of thermally activated dislocations and the possibility of grain boundary sliding [44,45,46,47,48].

A clear tendency towards higher yield strength values with increasing deformation degree was observed, as shown in Figure 2a. However, at 800 °C the values of all four material conditions nearly coincide. Comparing the undeformed specimens (φ = 0) with the lowest extruded condition (φ = 0.6), an enhancement of around 180 MPa in R_p0.2_ at RT is evident. This gain of strength is mainly attributed to the smaller grain size within the microstructure, as shown in Figure 1b, due to continuous dynamic and static recrystallization processes during hot-extrusion and the following HT. In the course of this process, the grain size of the (α_2_/γ)_col_ and γ_g_ is effectively reduced and the strength increases through the well-known Hall–Petch mechanism [35,36,49]. Especially below the brittle-to-ductile transition temperature (between 600 °C and 700 °C), the grain size dominates the strength properties [50].

As already mentioned, a further increase in the degree of deformation leads to a mean grain size of 1.8 μm (φ = 1.4 and φ = 1.9), as shown in Figure 1c,d. This refinement results in a yield strength of more than 1100 MPa at RT, which is dominated by the already discussed mechanisms. The specimens with φ = 1.4 exhibit better mechanical behavior in terms of R_p0.2_, UTS, and fracture strain (ε_F_) compared to the φ = 1.9 deformed specimens.

The lower average tensile properties of the φ = 1.9 specimens may be reasoned by the increased diameter of the starting ingot, which leads to slower cooling conditions during solidification. The starting ingot diameter must be increased to achieve the desired degree of deformation while keeping the final dimension almost constant. Hence, the occurrence of segregations, as well as an unsuitable microstructure, is assumed to become more likely within this condition [51]. This may be the reason for the lower average tensile values of the φ = 1.9 compared to the φ = 1.4 specimens.

Figure 2b shows the exemplary stress-strain (σ-ε) curves at temperatures from RT to 800 °C of the specimens with φ = 1.4. At RT, a maximum stress of 1221 MPa, as well as an ε_F_ value of 1.8%, could be obtained [7,9]. These high values are mainly attributed to the homogenous and fine-grained microstructure, as shown in Figure 1c. The cast/HIPed state exhibits at a temperature of 600 °C slightly higher ε_F_ values compared to RT tests [52]. This can be attributed to the enhanced possibility of dislocation motion and twin formation within the γ-phase leading to mitigation effects of local internal concentrations of stresses [15,45,48,52,53]. Hence, higher mechanical properties at RT are obtained when compared to the φ = 0 specimen. By increasing the temperature within the tensile tests from 600 °C to 700 °C, the brittle-to-ductile temperature is exceeded and a significant improvement of the fracture strain is observed. At a temperature of 800 °C, a maximum ε_F_ of about 87% occurred, which corresponds to a significant plastic deformability prior to failure [8,21]. However, the increasing fracture strain is accompanied by a decreasing R_p0.2_ and, thus, restricts the service temperature of the alloy.

### 3.3. Rotating Bending Tests

**Figure 3:**
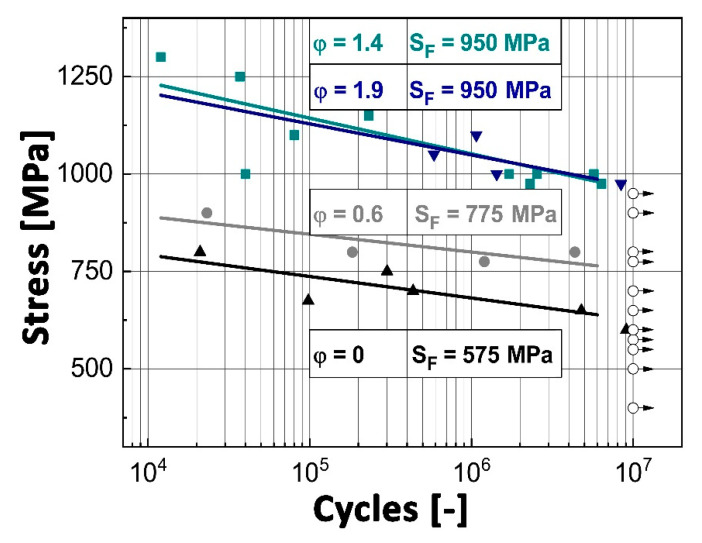
Results of the rotating bending tests conducted on different hot-extruded specimens at RT using a testing frequency of 100 Hz are displayed. Each dot represents the rupture of a specimen at the given stress amplitude. Open symbols with arrows indicate measured specimens where no fracture occurred until 10^7^ cycles.

To evaluate the effect of the deformation ratio on fatigue properties, rotating bending tests were conducted, and the results are also summarized in Table 1. With increasing φ, the fatigue limit, defined at 10^7^ cycles in this study, could be significantly enhanced until the minimum grain size at φ = 1.4 was reached. At the highest degree of deformation, the same fatigue strength (S_F_) of 950 MPa was obtained, because of the similar grain size. The stress (S) applied to the sample, plotted against the number of cycles (N) to fatigue failure, commonly called the S-N curve, is shown in Figure 3. The slopes of the interpolated linear S-N curves are flat, which is in good accordance with [54]. Within each experimental series of the differently deformed conditions, a relatively low scattering is observed. The similar behavior of the two highest deformed conditions is attributed to their almost identical microstructure, as shown in Figure 1c,d. At RT, the γ-phase is prone to micro-plastic deformation through dislocation movement [47,55,56,57]. The generated strain accumulation leads to crack initiation at the boundaries of the relatively soft and globular γ-grains [58,59]. Consequently, the coalescence of these early defects leads to the formation of a pre-crack at the grain boundaries, which propagates further by stable crack growth [56,58,60].

Taking into account the results of the tensile tests at RT and the determined fatigue limits, a proportionality factor ranging from 0.78 to 0.85, UTS, and S_F_ can be determined. This is in good accordance with the work of Sastry and Lipsitt [61] for fatigue experiments up to 10^6^ cycles, where the authors report a ratio of 0.8 at RT.

### 3.4. Fracture Analysis

**Figure 4:**
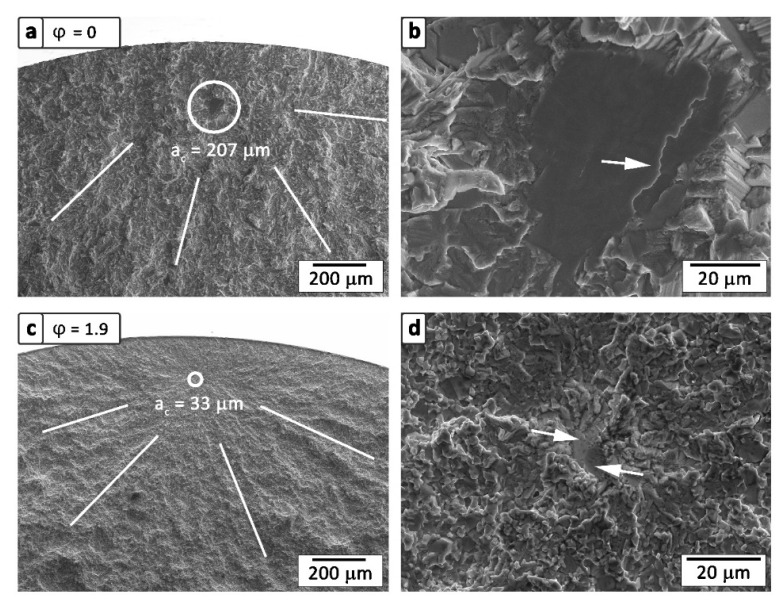
Fracture mirrors including the critical crack length a_c_ as well as crack initiation sites of selected, fractured rotating bending test specimens taken by SEM in secondary electron (SE) mode. A representative fracture surface of an undeformed specimen (**a**,**b**) appears to be rougher in contrast to the deformed specimen (**c**,**d**). It shows a cleaved (α_2_/γ)_col_ as an initial crack, including a translamellar step, which is marked by an arrow in (**b**). In (**c**,**d**), a hot-extruded sample with a degree of deformation of φ = 1.9 is depicted. Here, coarse γ-grains with different angles to the image plane act as initial cracks and are highlighted in (**d**) by white arrows.

The fractured surfaces of the specimens from rotating bending tests were analyzed with SEM using SE mode. Two different cases can be distinguished:

The undeformed samples (cast/HIPed and HT) show smooth initial cracks within the occurring fracture mirror, as shown in Figure 4a. These cracks typically possess a dimension comparable to the size of the present (α_2_/γ)_col_ and exhibit steps, which are exemplary marked by an arrow within Figure 4b. Thus, unfavorable orientated colonies were cleaved by interlamellar fracture [56,62]. Crack initiation was observed not to occur at surface defects, but at colonies located just below the sample surface. This is caused by the presence of compressive stress close to the specimens’ surface, which stems from the mechanical preparation process. The cleavage of the lamellae mainly takes place at the α_2_/γ-interfaces [63]. Steps, as visible in Figure 4b, occur when the crack path continues to a neighboring α_2_/γ-interface [64,65]. Thus, it is assumed that these steps connect pre-existent cracks in front of the crack tip. During stable crack growth, the surrounding colonies largely fail in a translamellar manner at higher crack growth rates in case of a random orientation distribution [64]. As described by Leitner et al. [66], the occurrence of a brittle β_o_-film surrounding the colonies may accelerate the failure of the specimen. However, the presence of globular γ and β_o_ grains along (α_2_/γ)_col_ is considered to enhance the fracture behavior as discussed in [62].

**Figure 5:**
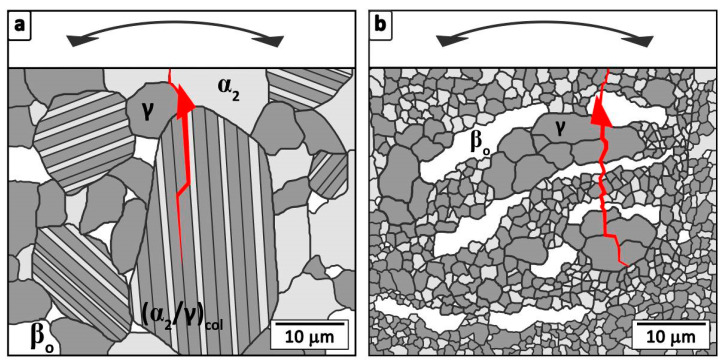
Schematic illustration of the two different predominant crack initiation and propagation modes. (**a**) Crack initiation (red triangle) at the interface between an α_2_- and γ-grain as well as the further crack growth within a cast/HIPed and heat treated microstructure. Subsequently, the crack splits a coarse (α_2_/γ)_col_, including inter- and translamellar propagation. (**b**) Crack growth within the hot-extruded material with a fine-grained microstructure. At RT, the crack initiation takes place below the specimen surface within the brittle elongated β_o_ phase and propagates preferentially through the coarse γ grains.

Crack propagation, including a step within an α_2_/γ-colony, is schematically depicted in Figure 5a. A red triangle symbolizes the initial crack below the surface at a γ-grain interface. Further crack growth through the coarse (α_2_/γ)_col_ is illustrated by the red line at the interfaces within the colony. Hence, the crack reaches the surface at the prevailing tensile stress, which the rotating bending specimen is subjected to.

The fracture appearance of the hot-extruded samples, as shown in Figure 4c,d, exhibit smaller initial cracks. These cracks are surrounded by a typical fracture mirror and show almost the same size, as shown in Figure 4c, as the already mentioned coarse globular γ-phase within Figure 1b,d. Here, the relatively soft γ-phase is assumed to deform, which enables the formation of micro-cracks within the adjacent brittle β_o_-phase, as schematically shown in Figure 5b. The initial crack is symbolized by a red triangle and the growing crack is depicted by a red line. After crack initiation, the crack grows almost unconstrained through the near γ_coarse_-grains, since its propagation is hardly hindered by crack deflection.

Once the crack reaches a critical length of a_c_, the stress intensity at the crack tip exceeds the material’s strength and the remaining cross-section fails by unstable crack propagation. Therefore, an assessment based on linear-elastic fracture mechanics according to Equation (2) can be conducted to allow an estimation of the critical conditional fracture toughness [67,68]:

(2) $$K_{\mathrm{IC}}\;=\;Y\;\cdot \;\sigma \;\cdot \;\sqrt{\pi \;\cdot {\;a}_{c}}$$

Here, the measured dimensions of the specimens’ fracture mirrors from rotating bending tests were used to calculate the conditional critical fracture toughness K_IC_ [67,68]. The geometrical constant Y is defined as 2/π and represents a penny-shaped inner crack under tensile loading. SEM investigations on the fractured specimens allowed the obtaining of the dimensions of the fracture mirrors, which correspond to a_c_, as shown in Figure 4a,c. Due to the experimental setup of rotational bending tests, the bending stress on the surfaces of the specimens is constant over the specimen’s length. Therefore, the specimens are assumed to fail at the maximal bending stress σ. It should be pointed out that the determined K_IC_ values are intended to be compared within this experimental study, but do not represent standardized K_IC_ values [68]. Therefore, only conditional K_IC_ values are discussed below. The mean K_IC_ values, including a standard deviation from at least three specimens, are listed in Table 1. The results indicate a higher resistance to crack propagation of the undeformed microstructure, exhibiting a fracture toughness of about 9.2 ± 0.7 $\mathrm{MPa}\sqrt{m}$, in comparison to the extruded fine-grained microstructures which show lower values [23,65,66,69,70]. Within the deformed samples, the calculated fracture toughness is significantly lower (6.5 ± 0.5 $\mathrm{MPa}\sqrt{m}$). The obtained fracture toughness values are in accordance with the results of Appel et al. [71] with 9.2 $\mathrm{MPa}\sqrt{m}$ and Leitner et al. [66] with 12.1 ± 0.7 $\mathrm{MPa}\sqrt{m}$ for similar TiAl alloys and material conditions. The decreasing fracture toughness of the hot-deformed specimens can be explained by the prevailing crack propagation mechanism. In the case of the fine-grained material conditions, the crack propagates easier through smaller globular grains than through a coarse-grained microstructure, where crack deflection and other toughening mechanisms prevail [70,72,73]. In this study, the specimens exhibiting a φ = 0.6 show a similar fracture toughness as the higher deformed samples despite the coarser globular γ-grains and persistent (α_2_/γ)_col_ present in this condition, as shown in Figure 1b. Here, the prevailing bimodal grain size distribution must be taken into account, including a fine-grained matrix as well as coarser microstructural constituents. Consequently, the material behaves like the φ = 1.4 and 1.9 hot-extruded materials in terms of fracture toughness. Therefore, crack nucleation and propagation are assumed to take place within the fine-grained matrix in the φ = 0.6 samples, which is comparable to case (ii), as previously discussed.

## 4. Conclusions

Within this study, the dependence of mechanical properties on microstructure in an engineering intermetallic γ-TiAl-based alloy was shown, against the background of obtaining optimized properties regarding high-performance racing applications, like inlet and outlet combustion valves. For this purpose, HIPed TNM ingots with a nominal composition of Ti–43.5Al–4Nb–1Mo–0.1B (in at%) were hot-extruded to different degrees of deformation and subsequently heat treated. The adjusted microstructures were assessed using tensile tests, rotating bending tests, and microstructural analysis. The major findings can be summarized as follows:

- With an increasing deformation ratio, a finer grain size can be obtained. This fact lasts until φ = 1.4, where a saturation level is reached with a grain size of about 1.8 μm. The characteristic α_2_/γ colonies disappear continuously and are replaced by globular α_2_- and γ-grains through dynamic and static recrystallization, surrounded by elongated β_o_-grains.
- Employing RT tensile tests, a fracture strain of about 1.3% and a yield strength of 1121 MPa are determined for φ = 1.4. This condition exhibits a fine-grained and homogenous microstructure. At 800 °C, the yield strength amounts to 404 MPa and a fracture strain of more than 87% can be obtained. Additionally, a decreasing grain size is observed, which results in a significantly lower brittle-to-ductile transition temperature.
- A clear dependence between the fatigue limit and the degree of deformation is found. Caused by the smaller grain size as a result of the extrusion process, the material’s fatigue limit at RT increases from 575 to 950 MPa. Here, large microstructural constituents are identified as initial defects within the microstructure. However, a high deformation degree reduces the size of these constituents, which is an effective way to increase the endurance limit. Comparing the ultimate tensile strength and the fatigue limit of the different deformed specimens allowed the determination of a proportionality factor in the range of 0.78 to 0.85.
- Two different crack initiation modes are determined depending on the existing microstructure. (i) Within the cast/HIPed and HT microstructure, the crack initiates at the boundaries of coarse (α_2_/γ)_col_ near the sample surface and cleaves the colony in an interlamellar way along the α_2_/γ-interfaces. (ii) Initial defects are identified as elongated and globular γ-grains in deformed and heat treated material conditions. Consequently, to attain a homogeneous and defect-free microstructure is pivotal for the fatigue behavior.
- A conditional fracture toughness can be estimated based on the dimensions of the so-called fracture mirrors of the failed fatigue samples. This value amounts to 9.2 ± 0.7 $\mathrm{MPa}\sqrt{m}$ for the undeformed and about 6.5 ± 0.5$\;\mathrm{MPa}\sqrt{m}$ in case of the differently deformed conditions.

Based on the results of tensile and rotating bending tests, the process of hot-extrusion is a promising method for the production of material conditions required in high-performance racing applications. The high fatigue limit and strength of the deformed TNM alloy are beneficial for applications with fast cyclic loading conditions. The fracture behavior indicates a high sensitivity of the investigated alloy system on the microstructure and prevailing inner defects. Therefore, the effect of grain refinement and homogenization during the deformation process provides promising and balanced mechanical properties.

## Figures and Tables

**Table 1:** Summary of the mechanical properties obtained for the TNM alloy at room temperature (RT), 600 °C, 700 °C, and 800 °C with varying φ, including the standard deviation of the respective data point. UTS: ultimate tensile strength.

φ	T	$\bar{d}$	HV10	UTS	R_p0.2_	ε_F_	S_F_	K_IC_^a^
[–]	[°C]	[μm]	[–]	[MPa]	[MPa]	[%]	[MPa]	$[\mathrm{MPa}\sqrt{m}$ **]**
0	25	10.7 ± 4.2	342 ± 3	675 ± 27	660 ± 11	0.2 ± 0.1	575	9.2 ± 0.7
	600	–	–	710 ± 2	568 ± 11	1.8 ± 0.3	–	–
	700	–	–	678 ± –	535 ± –	1.6 ± –	–	–
	800	–	–	549 ± –	439 ± –	22.6 ± –		–
0.6	25	6.9 ± 3.9	356 ± 3	929 ± 1	840 ± 8	1.7 ± 0.1	775	6.6 ± 0.4
	600	–	–	– ^b^	– ^b^	– ^b^	–	–
	700	–	–	– ^b^	– ^b^	– ^b^	–	–
	800	–	–	526 ± 0	433 ± 1	90.2 ± 21	–	–
1.4	25	1.8 ± 0.4	393 ± 2	1213 ± 11	1121 ± 0	1.3 ± 0.7	950	6.4 ± 0.5
	600	–	–	1009 ± 20	860 ± 8	1.7 ± 0.1	–	–
	700	–	–	863 ± 2	673 ± 1	61.4 ± 4.8	–	–
	800	–	–	568 ± 18	404 ± 11	87.4 ± 14	–	–
1.9	25	1.8 ± 0.5	386 ± 2	1133 ± 18	1108 ± 18	1.8 ± 0.1	950	6.5 ± 0.4
	600	–	–	917 ± 20	795 ± 21	2.5 ± 0.1	–	–
	700	–	–	794 ± 3	639 ± 6	52.2 ± 30	–	–
	800	–	–	528 ± 6	399 ± 8	80.6 ± 8	–	–

^a^ The conditional K_IC_ values were assessed using the area concept as described in Section 3.4. ^b^ No valid experimental values available due to the lack of sample material.
